# The Medical Benefits of Vitamin K_2_ on Calcium-Related Disorders

**DOI:** 10.3390/nu13020691

**Published:** 2021-02-21

**Authors:** Zeyad Khalil, Benyamin Alam, Amir Reza Akbari, Harbans Sharma

**Affiliations:** Medical School, The University of Manchester, Oxford Road, Manchester M13 9PL, UK; benyamin.alam@student.manchester.ac.uk (B.A.); amir.akbari@student.manchester.ac.uk (A.R.A.); harbans.sharma@manchester.ac.uk (H.S.)

**Keywords:** vitamin K_2_, vitamin D, calcium, metabolism, osteoporosis, cardiovascular disease, parathyroid

## Abstract

Background: Due to the potentially crucial role of vitamin K_2_ in calcium metabolism, a deficit can disrupt many mechanisms, resulting in an array of different issues, such as broken bones, stiff arteries and poor fertility. Although there has been existing research, the potential of vitamin K_2_ as a treatment for conditions including cerebral palsy, parathyroid disease, heart disease and gastrointestinal disease is unknown. This review discusses the biochemistry of vitamin K and the metabolism of calcium, followed by an analysis of the current literature available on vitamin K_2_ and its prospects. Methods: Using public libraries including PubMed and Wiley, we searched for existing research on the metabolism and use of vitamin K_2_ that has been conducted in the preceding two decades. Results: Data indicated that vitamin K_2_ had a positive impact on osteoporosis, cardiovascular disease, parathyroid disorders, cerebral palsy and sperm motility. Conclusion: Due to the existence of confounding variables and limitations in the quality and volume of research conducted, further investigation must be done to see whether the beneficial effects seen are reproducible and must assess the viability of vitamin K_2_ as treatment in isolation for these conditions.

## 1. Introduction

Vitamin K has undergone comprehensive research over a long period, revealing its array of medicinal properties that have proven beneficial, leading to its review for use in the medical community. It was first discovered by Henrik Dam in 1935; through experimenting the regimens of chickens and interchanging various sources of their diets, Dam isolated this compound and named it after the German word for coagulation. The chickens showed a decrease in haemorrhages when given regimens with the vitamin, and this was the defining moment that sparked further research into Vitamin K [1]. 

Following the initial identification, it was soon established that there are multiple types of vitamin K. It can be divided into vitamins K_1_, K_2_, K_3_, K_4_ and K_5_. The two major subtypes are vitamin K_1_ and K_2_, whereas the vitamin K_3_ is a synthetic form which can be converted to vitamin K_2_ in vivo. Contrastingly, vitamin K_1_ and K_2_ are both fat soluble allowing them to enter cells without the need of transmembrane transport proteins, particularly in animals [2], while K_3_ is water soluble [3]. It has been hypothesised that vitamin K_2_ is just as significant in effect as K_1_, owing to the differing structure of these subtypes.

Since this discovery, research has flourished on the topic, resulting in the collation of a large volume of information, allowing for a greater understanding of the vitamin. Evidence has elucidated its vital role in the coagulation pathway, with more recent research investigating the effect it has on extra-hepatic processes in the body. In particular, vitamin K_2_ has shown to have a considerable effect on calcium metabolism. The main batch of research in the past few years has been on how it effects the remodelling of bones, which yielded limited clinical application, though improved the understanding of this vitamin, as illustrated in the discovery of novel areas of benefit, such as the reduction of calcification of blood vessels within the body. 

Bone remodelling requires a variety of cells including osteoblasts, osteoclasts and osteocytes. Osteoblasts are constantly synthesising the non-collagenous protein osteocalcin. Osteocalcin is one of the most copious proteins in bone and has been proven to be one of numerous vitamin K-dependent proteins. This would make vitamin K essential in bone remodelling, one of its many roles in calcium metabolism [4].

Currently, there are 7 million people living with cardiovascular disease in the UK and the cost of their care is estimated to be £9 billion each year [5]. Many of these people will be undergoing treatment for diseases such as hypertension. These are a few cardiovascular diseases which can be caused by arterial calcification, especially of the major blood vessels such as the aorta. Vitamin K_2_ is effective in reducing the levels of calcium available in the blood, thereby decreasing the rate of deposition in vascular structures [6]. However, some studies researching this topic used other vitamins and minerals alongside vitamin K_2_, such as calcium and vitamin D. This could either represent a confounding variable decreasing reliability of the articles or suggest a synergistic effect of taking vitamin K_2_ with other vitamins. 

Vitamin K_2_ has a multitude of other effects on calcium due to the presence of vitamin K-dependent proteins. It remains unknown whether vitamin K_2_ in isolation is responsible for its perceived positive attributes. This narrative review will look further into the biochemistry behind the vitamin K and cover the metabolism of calcium before combining the two and comparing the current research to more basic studies. This will enable a more definitive opinion on the possible benefit of vitamin K_2_ on calcium metabolism.

## 2. Biochemistry of Vitamin K

Before looking at the specific effects that vitamin K_2_ establishes on calcium metabolism, it is vital to consider the fundamental properties that a vitamin possesses. They are organic compounds that are found in our natural diet. Vitamins have essential physiological functions within the body, and without sufficient levels, disease can arise. Each vitamin not only differs through their function but also in how they are metabolised in the body. For example, vitamin D can be synthesised through the interaction of ultraviolet B rays activating 7-dehydrocholesterol (7-DHC) which is present in the skin [7]. The precursor to this active form of the vitamin D is termed the vitamer, defined as a molecular analogue of its given vitamin. This feature of inter-changeability from their active and inactive forms is common amongst vitamins [1].

**Figure 1:**
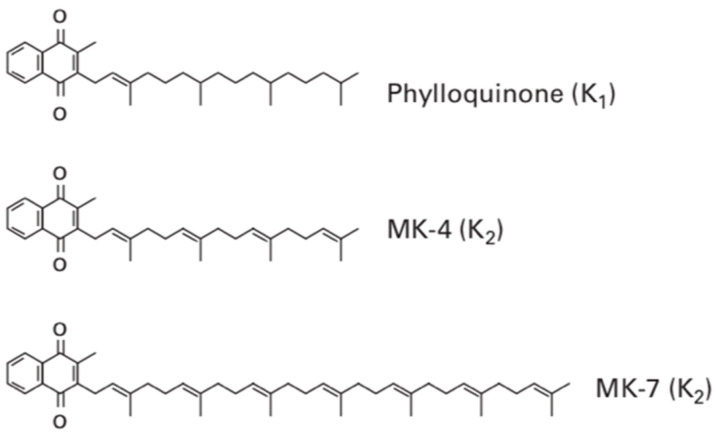
The chemical structure of Vitamin K. Phylloquinone is shown to contain repeating prenyl units, where it is bonded to the third carbon on the menadione ring. In comparison, MK-4 and MK-7 contain the same menadione ring, though it is the prenyl repeating units that bond to the third carbon. The length of the MK-7 chain is three units longer, which is what gives it a longer half-life compared to MK-4 [8].

Vitamin K contains several subtypes which all contain the fundamental 2-methyl-1,4-naphthoquinone ring structure also termed menadione. Vitamin K_1_ contains this menadione ring with a phytyl side chain present on the third carbon of the menadione structure. This makes it remarkably similar to the structure of chlorophyll, and therefore, it is commonly found in sources of green leafy vegetables. Vitamin K_2_, sometimes termed menaquinone, contrastingly has a varying length of side chain and rather than the phytyl side chain found in phylloquinone (vitamin K_1_), it contains repeating prenyl units, as shown in Figure 1. The number of repeating prenyl units in the side chain relates to the classification of vitamin K_2_, allowing further differentiation.

This allows menaquinones to be abbreviated to menaquinone-n (MK-n), where the n relates to the number of repeats of the prenyl units. The most commonly found menaquinones are MK-4 and MK-7; however, the number of lipophilic side chains can range from 4–14. Although vitamin K_3_, K_4_ and K_5_ exist, they are only available as a synthetic form [9]. Whilst vitamin K_3_ is often fed to chickens, which then convert this into vitamin K_2_ [10], the aforementioned variants of vitamin K will not be discussed in this report, as this is exclusively an investigation of the direct use of vitamin K on human patients.

The source of both major forms of vitamin K differs; vitamin K_1_ is found most predominantly in green leafy vegetables, such as spinach, cabbage and kale, and absorption is increased in the presence of butter or oils. [9]. Sources of vitamin K_2_ differ with the variation of their menaquinone lengths. MK-4 is found within animal products, such as chicken meat, beef and salmon. The richest source of MK-7 comes from bacterially fermented food, such as natto, a traditional soybean dish commonly found in Japan. The production of natto is challenging due to the specific measurements required for the beans and the difficult fermentation process [11]; this perhaps explains the scarcity of this type of nutrition in other countries’ diets. This increased vitamin K_2_ intake found in the diet of the Japanese population is what lead to the founding research studies into its beneficial effects on calcium metabolism, especially as it was correlated to reduced incidence of osteoporosis [12]. Research is being conducted to clarify whether the Western diet currently contains a viable amount of the vitamin K_2_, as it is only found in particular foods. Other sources of food rich in vitamin K_2_ include egg yolks, hard cheeses, cottage cheese, butter and sauerkraut. As both vitamin K_1_ and K_2_ are produced by plants, they are both commonly found in the intestinal tract due to the presence of fermenting bacteria. Bacteria such as *Escherichia coli* are capable of manufacturing vitamin K_2_; however, they only produce the menaquinones MK-7 to MK-11 [1]. *Bacilus Subtilis*, another fermenting bacterium, is responsible for the high levels of MK-7 present in natto with many other bacteria; however, it has only been identified in the colon, where there is limited absorption of nutrients—the impact of these bacteria is currently undergoing further research [13].

In vivo, vitamin K is absorbed via enterocytes of the small intestine. This process is aided by the presence of bile salts and secretions from the pancreas. Upon absorption, they are packaged in vesicles named chylomicrons and secreted into the lacteal structures present in the enterocytes through exocytosis, a process that can be disturbed in many hospitalised patients [2]. These chylomicrons containing the fat-soluble vitamin K are released into the blood through the thoracic duct, into the left subclavian vein [14]. One study showed that plasma levels of phylloquinone peaked at 6 h after a meal. Furthermore, 75–90% of the ingested vitamin K was still present in the triglyceride-rich lipoproteins (TRLs), with the rest being transported by both high-density lipoproteins (HDLs) and low-density lipoproteins (LDLs) equally. In hepatic tissue, apoproteins such as apoE assist the binding of lipoproteins to their receptors, therefore acting as a ligand for internal uptake of the remnants of said lipoproteins. It is this same mechanism that causes uptake of vitamin K in hepatic tissue, which is evident through the detection of the breakdown products in both bile and urine [15]. Research into vitamin K uptake in bone revealed similar findings of increased levels of apoE, which suggests the presence of lipoprotein receptors on osteoblasts. This correlated with a decrease in the levels of uncarboxylated osteocalcin (ucOC), meaning vitamin K must have been actively absorbed into the bone matrix [16]. Another cell-surface protein called heparan sulphate proteoglycan (HSPG) plays a role in vitamin K uptake in bone. One study found that removing these from the surface caused a reduction in phylloquinone uptake [15]. 

**Figure 2:**
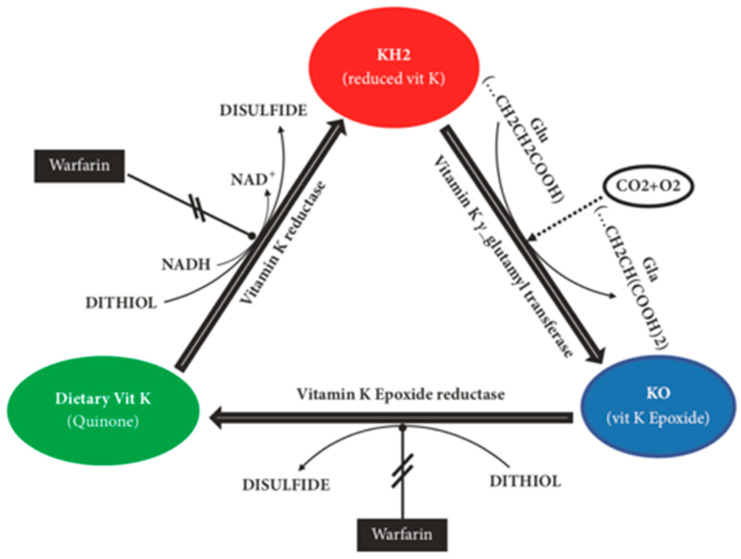
The vitamin K cycle. The initial step in the cycle is for the enzyme vitamin K reductase to convert the dietary vitamin K into reduced vitamin K (KH_2_). The enzyme vitamin K γ-glutamyl transferase then converts this into vitamin K epoxide (KO), which activates the compound. Finally, this is reduced back into the dietary form of vitamin K by the enzyme vitamin E epoxide reductase. Warfarin can halt the actions of this enzyme as well as the vitamin K reductase, enabling prolongation of the International Normalised Ratio (INR) [3].

Initially, vitamin K was solely associated to benefits in the coagulation cascade through interactions with numerous vitamin K-dependent proteins (VKDP), such as prothrombin (II), proconvertin (VII), antihaemophilia factor (IX), Stuart factor (X) and anticoagulant proteins C, S and Z. Through further research, it became clear that vitamin K had a more significant effect than previously thought, being a vital activating factor for many more VKDPs, such as matrix Gla protein (MGP), growth arrest-specific protein 6 (Gas6), osteocalcin (OC), Gla-rich protein (GRP), periostin, periostin-like factor (PLF), proline-rich Gla protein (PRGP) 1, PRGP2, transmembrane Gla protein (TMG) 3 and TMG4 [17]. Vitamin K_1_ and K_2_ are cofactors for the enzyme γ-glutamyl carboxylase (GGCX) and aid in the post-translational carboxylation of protein-bound glutamate residues into γ-carboxyglutamate. This is also known as Gla, and the aforementioned VKDPs are all members of this group of proteins, requiring both vitamin K_1_ and K_2_ to become carboxylated and carry out their individual functions [8]. Vitamin K must first be converted into vitamin K-hydroquinone (KH_2_) through a reduction reaction where this is then oxidised to vitamin K-epoxide (KO). The enzyme vitamin K-oxidoreductase (VKOR) converts this KO back into vitamin K restarting the entire process known as the vitamin K epoxide cycle, which is summarised in Figure 2 [9].

While vitamin K_1_ and K_2_ are structurally and functionally similar, they are metabolised in different ways. Vitamin K_1_ is the main constituent of vitamin K consumed in a Western diet, accounting for 75–90%, with the remainder owing to K_2_. Of the vitamin K_2_ absorbed, MK-4 accounts for 30–40%, with the rest absorbed from the longer chain menaquinones, such as MK-7, MK-8 and MK-9 [14]. The differing percentages between these sub-types of vitamin K_2_ could be caused by numerous variables, such as the presence of microflora such as *Escherichia coli*, which aids the synthesis of MK-7 to MK-11 in the large intestine [1]. This could also be due to MK-4 having a shorter half-life than the longer chain menaquinones [3]. The biological activity of vitamin K_1_ is favoured in coagulation, and therefore, is present in higher volumes in the liver; vitamin K_2_ also acts on coagulation, though it is more widely distributed within the body, being found in areas including the brain, pancreas, bones and genitalia [14].

## 3. Metabolism of Calcium

Calcium is directly influenced by the amounts of vitamin K in the body as it affects processes, such as the calcification of blood vessels, maturation of sperm in the testes and bone formation. When calcium metabolism is impaired, the resultant increase in arterial calcification and decreased calcium content of bone is known as the calcium paradox [13]. Calcium is predominantly regulated by parathyroid hormone (PTH) and vitamin D with other receptors for ionised calcium providing additional modulation. A decrease in serum calcium would be regulated through a negative feedback loop, which will result in reduced activation of calcium receptors in the parathyroid gland. The subsequent secretion of PTH causes increased reabsorption of calcium from the kidneys, while also increasing serum calcium through the activation of osteoclasts in the bone. The kidneys are also stimulated by PTH to secrete vitamin D, which allows more calcium to be taken up through calcium channels in the gastrointestinal tract (GIT) [18]. One Korean study illustrated that when vitamin D and calcium are supplemented with vitamin K, there was significant improvement in the bone mineral density (BMD) of postmenopausal women aged over 60 years old [19]. 

Abnormalities in calcium homeostasis can lead to an imbalance in the normal physiological functions of calcium. For example, in hypercalcaemia, the increased serum levels of calcium lead to a higher cardiac output, as calcium is used intracellularly for muscle contraction. Without the normal serum levels of calcium, 4.4 to 5.4 mg/dL, the body aims to correct this through increasing bone resorption which can weaken bones, while the kidneys attempt to compensate for this through excretion, causing dehydration [18]. 

Vascular calcification, a common pathology, damages arteries due to poor calcium regulation. Blood vessels consist of three layers, intima, media and adventitia. A layer called the internal elastic lamina separates the layer of endothelial cells of the intima from the smooth muscle cells (SMCs) of the media. The external elastic lamina separates the media from the adventitia; arterioles which supply the SMCs in larger vessels can be found within this lamina. The adventitia contains nerve fibres which react to signals, inducing vasoconstriction or vasodilation in the SMCs. The SMCs synthesise elastin, collagen, proteoglycans, cytokines and growth factors. These migrate to the intima during vascular injury and can aid in the calcification of arteries [20].

**Figure 3:**
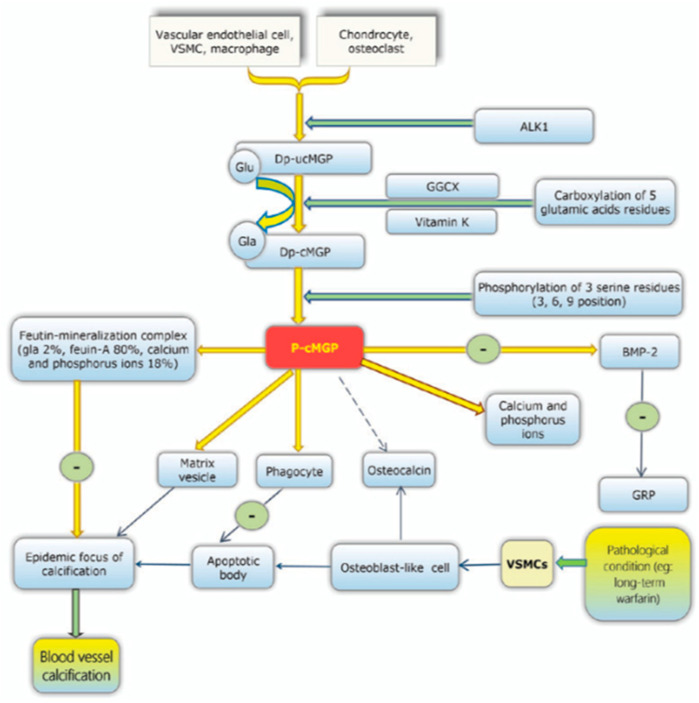
Progression of vascular calcification. This process highlights the importance of carboxylated MGP in its ability to adopt its properties once vitamin K and GGCX do so. However, once the MGP protein becomes phosphorylated, it then has specific physiological functions, such as inducing phagocytes to remove apoptotic bodies, chelating to calcium and phosphorus ions and stimulating the production of osteocalcin, which all prevent the calcification of vessels. Bone Mineral protein (BMP), Gla-rich protein (GRP), Vascular smooth muscle cells (VSMCs) [17].

Vascular calcification is a result of the SMCs behaving like osteoblasts, allowing synthesis hydroxyapatite crystals, similar to the bone remodelling process. This occurs in the media layer and as a result the artery becomes stiff and unable to dilate or constrict [21]. The underlying pathology is due to a lack of activation of the inhibitory proteins that prevent this mineralisation of the vessels. MGP is one such protein and functions to inhibit the deposition of hydroxyapatite crystals preventing vascular calcification. This is one of many VKDPs which require vitamin K to act as a co-factor in its activation. In the case of MGP, dephosphorylated- uncarboxylated MGP (dp-ucMGP) is phosphorylated into undercarboxylated MGP (ucMGP). This allows further carboxylation of five glutamate residues by the active form of vitamin K and its co-factor GGCX. These residues provide active sites for the removal of apoptotic bodies, calcium ions and matrix vesicles [17]. Other VKDPs involved in the prevention of vascular calcification include GRP, periostin, Gas6, OC, TMG 3, TMG 4, PRGP 1, PRGP 2 and PLF [8]. See Figure 3. 

MGP can also be activated through the phosphorylation of three serine residues, which results in the negatively charged phosphate group binding to the calcified formation, preventing further calcification. Bone morphogenetic proteins (BMP) 2 and BMP 4 have been hypothesised to be inhibited by MGP. Normally, BMP-2 induces apoptosis, thereby enhancing the calcification process. Carboxylated MGP is less active due to a reduced vitamin K_2_ intake, this leads to a higher expression of BMP-2 leading to more deposition of the hydroxyapatite crystals [22]. 

**Figure 4:**
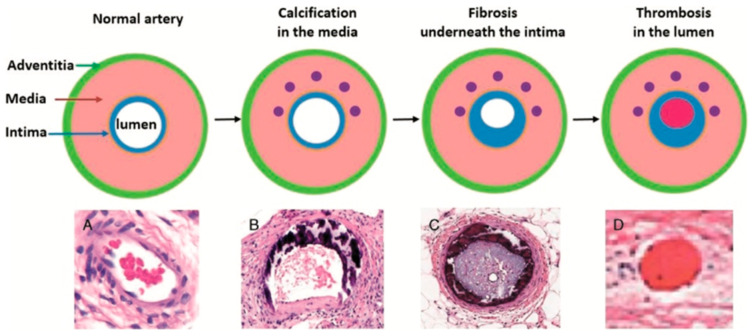
Histological progression of calcification. Initially, the calcification occurs in the media of the arteries due to the presence of uncarboxylated MGP alongside other factors such as apoptotic bodies, calcium ions, phosphate ions and matrix vesicles. Together, these form the initial subendothelial deposition, which contains hydroxyapatite crystals that then continue to mineralise and occlude the lumen of the vessel. Lipids can then deposit on top of the calcified surface of the atherosclerotic plaque continuing to grow into the lumen. This entire deposition can then break off and form a thrombus, which can lead to strokes, myocardial infarction and other cardiovascular disease [21]. Arterial vessels contain 3 layers: the adventia, media and intima enclosing the lumen. Initially there is no calcification in these vessels (**A**). Over time calcium deposits accumulate in the media; this is common in many people and unlikely to cause pathology (**B**). As further calcium accumulates, new deposits develop underneath the intima, causing obstruc-tion of blood flow (**C**). Obstruction can cause turbulent blood flow and a hypercoagulable state, leading to thrombosis within the lumen (**D**).

Many conditions are closely related to vascular calcification, including hyperlipidaemia, chronic kidney disease (CKD) and diabetes. These diseases can increase the risk of calcification and formation of atherosclerotic plaques. This process is illustrated in Figure 4. One study on haemodialysis patients showed an increase in MK-7, which resulted in reduced levels of uncarboxylated osteocalcin and MGP, but only at a sufficient intake of vitamin K_2_ [23]. On the contrary, vitamin K_1_ does not have the same beneficial effect on reducing vascular calcification due to the LDLs transporting the majority of extra-hepatic vitamin K as the menaquinone subtype [22]. 

Calcium has multiple physiological functions; while vascular calcification is a common example, there are further diseases affecting bone. Stromal and mesenchymal stem cells are precursor cells found in bone, developing into osteoblasts and osteoclasts that carry out bone remodelling. These precursor cells line the active bone surface, helping to form the hydroxyapatite crystals in the bone matrix. Originating from the bone marrow, the cells will carry vitamin K_2_ and small amounts of vitamin K_1_, which they absorb from circulating stores in the blood [24]. 

The bone remodelling cycle occurs constantly in different phases around the body, with as its aim to strengthen the bone and repair any microfractures present. This cycle takes around 120 days to complete and is divided into six phases: quiescence, activation, resorption, reversal, early formation and late formation, before returning to the quiescence phase. Initially, the collagenous bone surface is removed, and the lining cells are retracted. During resorption, osteoclasts cleave the bone, leading to lacunae forming, after which they undergo apoptosis. This is when the osteoblasts become activated and monocytes and endosteal lining cells remove the debris from the previous phase. In the early formation phase, the osteoblasts produce the bone matrix, which is mineralised in the late formation phase, with the longest phase lasting 2 months or more [25]. The importance of vitamin D in calcium homeostasis must be stressed. It is responsible for many key roles in maintaining homeostasis, including promoting the cells in the bone for use in the remodelling cycle and reducing the apoptosis of osteoblasts; this promotes mineralisation and reduces the risk of fracture through protecting the trabecular bone [25]. 

Osteoporosis is one such metabolic bone disease where the bones weaken and are at increased risk of fracture. It is common in the UK, being estimated to cause 536,000 fractures [26]. Worldwide, it is believed that 1 in 3 women and 1 in 5 men over the age of 50 years are expected to experience an osteoporotic fracture [27]. Primary osteoporosis occurs in patients with postmenopausal oestrogen deficiency or aging in both genders, while secondary osteoporosis is due to nutritional deficiency and can also be caused iatrogenically through medication [27]. Research into osteoporosis on female patients showed a significant increase in the BMD of the test subjects supplemented with vitamin K_2_. This correlated with an increase in osteogenic activity, therefore providing evidence that the risk of osteoporotic fracture can be reduced through nutritional supplements, specifically vitamin K_2_. However, the effect of vitamin K_2_ on increasing BMD and reduced osteoclast activity was relatively small when compared to other standard medications, such as strontium ranelate of the supplements used in the study; strontium ranelate was shown to express the highest increase in BMD while also reducing osteoclast activity [28].

Primary hyperparathyroidism (PHP) is the result of increased serum levels of PTH. This can be from the parathyroid glands becoming hyperplasic and over-active or from ectopic sources, but the resulting effect in all cases is hypercalcaemia. The increase in serum calcium occurs due to the resorption of bone through the increase of both osteoclast activity calcium absorption from the GIT. Consequently, the BMD of individuals with PHP decreases in cortical-rich sites, such as the forearm. On the contrary, BMD in areas of cancellous bone such as in the spine and femoral neck are not as affected [27]. Currently, there is no research into whether vitamin K_2_ could be used as a treatment for patients with PHP to reduce the risk of vascular calcification and the resorption of bone during this phase of hypercalcaemia. The only treatment available for PHP is a parathyroidectomy [27]. 

Another function of calcium that remains under further study is its metabolism in the testes. Calcium ions aid in the development and activation of spermatozoa in the testes as they develop. Once released into the semen, calcium ions further contribute to their development, aiding in the stimulation of their mobility and improving the probability of fertilisation. Research has shown that calcium ions (Ca^2+^) regulate these spermatozoa through Ca^2+^ protein channels in the membrane of the reproductive cells [29]. Confirming that Ca^2+^ are essential for male fertility, one study has shown that vitamin D deficiency caused reduced sperm motility [30]. The lack of vitamin D results in decreased quantities of serum ionised calcium levels as well as reduced Ca^2+^ in the seminal fluid. Subsequently, this affects the vitamin D regulated calcium protein channels in the plasma membrane of the spermatozoa. Transmembrane protein channels were identified to facilitate the influx of Ca^2+^ into the spermatozoa. Cationic sperm (CatSper) channels 1 and 2 are transmembrane protein channels critical for sperm activation and motility, as revealed in one study where male participants with reduced sperm motility showed a reduction in CatSper 1 [31]. VKDPs GGCX and MGP were found to aid in the maturation of sperm in the epididymis. MGP concentrations were tenfold in the epididymis, providing insight into the effect of reduced levels of activated VKDPs on sperm maturation. After administration of warfarin to inhibit the GGCX enzyme, computer analysis showed a decrease in the percentage of total and progressively motile spermatozoa [32]. This further demonstrates an area of calcium metabolism within the body that could require further experimentation to determine the significance of vitamin K_2_ in sperm maturation and motility post ejaculation.

## 4. Analysing Current Literature

In this narrative review, we have accumulated previous studies conducted into vitamin K_2_ and have ordered them chronologically to give insight into how understanding has progressed. We have assessed multiple different types of reviews ranging from literature reviews to randomised control studies.

In Japan, it was established that the traditionally eaten natto was high in long chain menaquinones, which aided in a reduction in the rate of BMD loss. The first research on vitamin K_2_ was carried out in Japan in 1992 by Akedo et al. They conducted an experimental study, proposing Vitamin K_2_ both acts as a co-factor for γ-carboxylation of osteogenic cells and is activated itself by this process [33]. 

In 1996, Kameda et al. investigated the effect both vitamin K_1_ and K_2_ had on osteoclasts. The experimental study indicated that K_2_ inhibits pit formation in the bone; increasing dosage of K_2_ did not alter the number of pits but reduced their surface area. Additionally, vitamin K_2_ alone increased the rate of apoptosis of osteoclasts, with the study proposing that VKDP MGP mediates these effects on bone metabolism [34].

Another study in 2001 by Yamaguchi et al. experimented on the osteoblastic activity of rat bone cells that resulted from stimulation by MK-7. This resulted in a cellular increase of calcium in femoral-diaphyseal cells and a significant increase in protein synthesis of osteoblastic cells [35]. 

Shiraki et al. investigated use of the vitamer to treat 241 female osteoporotic patients, finding that serum levels of undercarboxylated osteocalcin were reduced after two years, while BMD density being maintained throughout. The incidence of new fractures in the group treated with MK-4 was decreased, but the paper concluded that further research was required to confirm the hypothesis [36]. 

In a randomised control trial in 2001, Shiomi et al. administered MK-4 to female Japanese patients with liver cirrhosis, and in the first year after treatment, they showed increased BMD; however, this returned to baseline value after two years [37]. 

In 2001, Kaneki et al. conducted a cross-sectional study to compare the serum vitamin K_2_ levels in Asian and European women. There were 31 British participants, 49 Eastern Japanese and 25 Western Japanese. The presence of natto in the diet of the Japanese population was responsible a marked increase in the serum levels of MK-7. As a result, the elevated serum level of vitamin K_2_ was sustained for longer than a single oral dose of MK-4 after a 24-h period. This may be partly due to the presence of fermenting bacteria in the intestine, which, combined with the intake of MK-7, resulted in a rise in serum vitamin K_2_. This study found that post-menopausal Japanese women had a reduced osteoporotic fracture risk compared to the aforementioned regions. A Spearman’s rank coefficient of −0.321 was displayed when assessing hip fracture incidence against familial expense of natto [12]. Scarcity of fermented foods rich in MK-7, such as natto, in the Western diet may be responsible for these results.

In 2002, Ozuru et al. set out to establish the effect of vitamin K_2_ by measuring the biochemical response in a prospective cohort study. After receiving the vitamin, bone biomarkers, such as carboxylated and uncarboxylated osteocalcin, and BMD were assessed. The study suggested a correlation between K_2_ and bone health. One month following administration, serum levels of carboxylated osteocalcin were elevated; however, minimal change was seen in BMD. This contradicts the results found in Shiomi et al., where BMD increased in the first year [38]. It is important to note that Ozuru et al. only studied a limited sample of 34 postmenopausal Japanese women, demonstrating a potential selection bias which would decrease the studies reliability. 

As the understanding of the role of vitamin K_2_ in calcium metabolism and bone mineralisation increased, further research set out to compare its effects with related compounds. A literature review in Tokyo by Iwamoto et al. investigated the treatment of postmenopausal osteoporosis with vitamin K_2_, vitamin D or both combined. It concluded that the synergistic effect of using both vitamin D and K_2_ was only present in young patients or mild cases of osteoporosis [39]. 

Taira et al. investigated the specific effects of vitamin K_2_ on individual cells involved in bone metabolism in an experimental study. They measured the area of lacunar resorption after administration of both vitamin K_1_ and K_2_. The results showed that vitamin K_1_ had no effect on osteoclast activity, supporting earlier research carried out by Kameda et al. in 1996. Conversely, vitamin K_2_ decreased the area of lacunar resorption; further analysis revealed side chains containing prenyl units were responsible for inhibiting the differentiation of monocytes to osteoblasts [40]. 

In 2005, Sakamoto et al. acknowledged that vitamin K_2_ possesses properties which prevent osteoporosis, prompting an investigation into the physiological process responsible for this attribute. This animal study examined the mechanism allowing menaquinone to act as a replacement antioxidant for oestrogen, by comparing older female rats to younger ones. The results contradicted the hypothesis, as there was no significant increase in the serum levels of antioxidants. In contrast to some of the prior research mentioned in this review, this study found no effect on serum levels of osteocalcin, indicating that bone mineralisation was not increased. This questions whether vitamin K_2_ is of any benefit in treating osteoporosis and if so, if it is only able to prevent the incidence of fractures at mild levels, as reported by Kobayshi et al. [39,41]. However, the significance of this study is uncertain, as it was conducted on animal subjects rather than humans.

The aforementioned research piqued the attention of the International Osteoporosis Foundation (IOF), leading Knapen et al. to conduct a randomised control trial. The study controlled many confounding variables, including subjects with a history of anti-coagulant therapy, hormone replacement therapy or any further treatment that could interfere with the functionality of vitamin K_2_. To measure the potential efficacy of vitamin K_2_ on postmenopausal bone strength, the subjects had dual energy X-ray absorptiometry (DXA) scans carried out to measure the BMD as well as measurement of serum levels of bone metabolic biomarkers. The results showed no significant differences in BMD or bone mineral content (BMC) between the group given MK-4. However, the MK-4 group did specifically show an increase in the BMD of the femoral neck and a decrease in the BMD of the lumbar vertebrae L2–L4. Serum levels of carboxylated osteocalcin marginally increased over the placebo group, while the ucOC serum levels remained constant in both groups. They acknowledged the synergistic effect it can have when given with vitamin D, which supports the results found by Kobayshi et al. Although the research was carried out with the use of MK-4, the IOF gave MK-7 as an alternative due to the longer half-life, which would allow a lower intake of vitamin K_2_, but increased and prolonged serum levels [42].

There has now been a multitude of studies revealing a synergistic effect of vitamin K_2_ and vitamin D. Miyake et al. studied this direct relationship between the two vitamins and how they affect osteoblasts. The study investigated the activity of the enzyme epoxidase in the vitamin K cycle and altered variables in the cellular environment to analyse the impact on osteoblasts. The epoxidase showed a significant increase in activity in the presence of vitamin D. The results of this study give insight into the mechanism behind the synergistic effect vitamin D and vitamin K_2_ have on bone mineralisation. It is evident that vitamin D also causes an increase in the carboxylation of OC, which, in combination with the action of vitamin K_2_, indicates vitamin D could be equally crucial in treatment of bone pathologies [43].

Shearer et al. conducted a literature review of the advantages of vitamin K. One issue raised with the results of prior experiments is that they could be skewed by the presence of vitamin K_2_-fermenting bacteria in the GI tract. Therefore, when the subjects of studies are given additional supplements, they should be isotopically marked to increase sensitivity [15]. In 2011, a study of 78 postmenopausal Korean women investigated the effects of administering 15 mg of vitamin K_2_ three times daily. After 6 months of treatment, the lumbar BMD of the group receiving vitamin K_2_ had increased significantly. The concentration of ucOC decreased, compared to no change in the control group, agreeing with the findings of Shiraki et al. [19,36]. This study revealed new information about the possible effects of vitamin K_2_ potentially decreasing triglyceride concentrations. This has not been previously reported, but it was known that triglycerides are involved in the intestinal transportation of vitamin K_2_ to target cells [19].

Huang et al. performed a large meta-analysis of all the current research, looking specifically at randomised controlled trials prior to 2014. The results being analysed included any significant changes in BMD, ucOC concentration and OC concentration; however, only 19 of the 811 titles initially drafted were viable for analysis. These articles did not meet the inclusion criteria because of varying factors, such as only focusing on vitamin K_1_, subjects having conflicting pathologies, non-randomised control studies and insufficient subject numbers. Six studies showed increased BMD in the groups administered vitamin K_2_, with 10 studies showing that over a prolonged period there was maintenance or subtle increases in the lumbar BMD. However, when looking at the BMD at the hip of both the control group and the vitamin K_2_ group, there was no significant change [44]. This contrasts the study carried out by the IOF, where the bone density of the hip was found to be increased, but the lumbar BMD had slightly decreased [42]. This meta-analysis also included randomised control trials where subjects did not have any bone pathologies, providing insight into how vitamin K_2_ could be used outside of treatment for osteoporosis. Two studies included in the review analysed the incidence of fractures of the subjects after being given vitamin K_2_, with no significant change being found. However, four studies conducted on osteoporotic patients displayed a significant decrease in the incidence of fractures in the vitamin K_2_ group, when compared to the control groups [44].

These results correlated with previous studies such as those conducted by Shiraki et al., confirming the application of vitamin K_2_ as a potential treatment for osteoporosis, alongside other management options. This is due to the significant decrease in the ucOC when compared to the control group in 6 of the 19 studies evaluated. There was some contradiction with long term ucOC, as two studies out of the nine investigating this did not find a significant difference in their control and variable groups. The other seven studies showed a total 52.8% decrease in ucOC, which suggests that bone health improved in osteoporotic patients, supporting the use of vitamin K_2_ as a potential treatment. While there were increases in ucOC, in the same studies, there was no variation in BMD; however, these studies included subjects without pathological bone disease [44]. This could indicate that vitamin K_2_ can be used as a treatment but has no prophylactic use. 

The BMD of the lumbar vertebrae, hip and head of the femur have been used as a marker for the effect of vitamin K_2_ on bone in most papers. Some studies have also implemented assay techniques such as ELISA to assess serum levels of OC in their subjects, with most of these studies revealing that vitamin K_2_ aided in reducing the concentration of ucOC in the blood. In 2015, Inaba et al. studied the direct effect of a daily intake of MK-7 on the levels of carboxylated OC. Unlike previous research, this study was able to directly compare the results between a group of postmenopausal women and a second group of healthy subjects aged 20–69 years old both given varying dosages of vitamin K_2_. Postmenopausal subjects were divided into four groups, with dosages increasing at 50 micrograms from 0 to 200. The second group of subjects were randomly split into two groups with one group being given a placebo and the other being given 100 micrograms of MK-7. The initial analysis of results in the postmenopausal group of women showed a marked increase in ucOC concentration in the group administered 0 micrograms of MK-7, which correlates to a significant decrease in OC the same concentration. Only at high doses of 100 and 200 micrograms was the ratio of carboxylated OC to ucOC significantly higher. Globally, there is no daily recommended dosage of vitamin K_2_, but in Japan, the recommended intake in 2010 was 60–75 micrograms—this was later raised to 150 micrograms in 2015. These studies could provide evidence for utilising the correct dose to increase the γ-carboxylation of VKDPs, such as OC, which could aid in forming a recommended dosage for use outside of Japan. [45]. In the second group of healthy 20 to 69-year-old subjects, the group given 100 micrograms of MK-7 showed an initial serum increase, which plateaued and then returned to the original value [45]. Evidently, there is only a short-term effect of administering MK-7 to healthy individuals. 

Although this research has been conducted to investigate the use of vitamin K_2_ in maintaining bone health, there have been further studies in recent years that explored its other functions. Vitamin K_2_ has been known to aid in the prevention of calcification of vessels in the body. Previous research such as a study by Shanahan et al. explored the expression of genes encoding the proteins involved in this process. Osteopontin (OP) is one such glycoprotein which is found in bone, where it aids in adhesion of osteoclasts to the bone matrix during bone resorption, regulates apoptosis and signals cells to sites of inflammation. During this prospective cohort study on the coronary artery sections of healthy individuals, there was no presence of mRNA for OP. This contrasts the other 13 of the 18 samples that had high levels of OP mRNA, although this was only in the parts of the sections where a select few macrophages expressed the gene. MGP was also examined in this study, and, unlike the outcome of OP, MGP mRNA was found in numerous cells, such as the vascular smooth muscle cells (VSMCs) of the medial and intimal vascular layers, macrophages and endothelial cells. This proposes that MGP is a large contributor to the calcification and fibrosis of vessels that can lead to CVD disease when in its uncarboxylated form [46].

Osteopontin and MGP are both VKDPs requiring sufficient volumes of vitamin K; scarcity of this vitamin will allow uncarboxylated Glu resides to interfere with the aforementioned VKDPs. Once activated through γ-carboxylation in the bone, hydroxyapatite crystals are deposited—as required in bone mineralisation. This study illustrated that a lack of carboxylation of MGP results in reduced inhibition of the calcification of the vessel, as MGP can chelate the calcium and phosphate ions. In addition to requiring vitamin K, these proteins also need the active form of vitamin D, as it binds to the promoter region of their genes and in turn results in transcription of the VKDPs [46]. 

Jiang et al. set out to provide further evidence for the importance of maintaining a high serum level of vitamin K_2_ in 2016. The aortas of Sprague Dawley rats were investigated, having been grouped by treatment dose of warfarin, and fed the same diet for weeks. The administered warfarin prevented vitamin K_1_ and K_2_ from aiding in the co-activation of VKDPs, to induce calcification of their aortas. In the rats that were given warfarin and then administered vitamin K_2_, there was a significant decrease in the calcification of their aortas, indicating a reversal of calcification. Within the same group of rats, the rate of apoptosis of the VSMCs decreased when given vitamin K_2_ six weeks after a diet containing warfarin. Gas6 is another VKDP that regulates the apoptosis of VSMCs; when both this protein and MGP are undercarboxylated, there is an increase in the rate of apoptosis. This study reveals the role of vitamin K_2_ not only as a co-factor in the activation of VKDPs, but also reversing the deposition of calcium in vessels. A key enzyme that regulates bone mineralisation, alkaline phosphatase (ALP), was found to have a higher activity when the warfarin was administered to the rats. Once the warfarin had been stopped, levels of ALP continued to increase until vitamin K_2_ was included in the rats’ diet. This provides insight into the different mechanisms that together form the calcified plaques found in the aorta of the rats; future research into how ALP becomes activated outside the bone matrix would prove useful [47].

Yi-Chou Hou et al. reviewed the synergistic effect of vitamins D and K in the physiological pathway of calcification of arteries in CKD. First, the review acknowledges that phosphorylated MGP inhibits calcification of vessels through binding directly to calcium and BMP-2. This prevents the VSMCs from differentiating into osteoblasts, therefore preventing apoptosis. Furthermore, administration of vitamin K_2_ caused a decreased level of ucOC, promoting bone mineralisation. As a result, there is less calcium and phosphate freely available in the blood, reducing the risk of vascular calcification.

**Figure 5:**
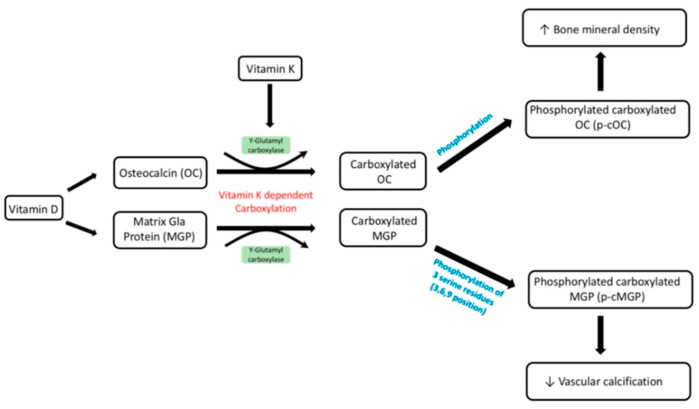
Metabolism of vitamin D and K. The active form of vitamin D can act as a transcription activator for the synthesis of OC and MGP. These are then carboxylated through the co-factors γ-glutamyl carboxylase and vitamin K. These proteins need to be further phosphorylated to enable normal physiological functions shown above, including phosphorylated carboxylated OC resulting in increased bone mineral density and phosphorylated carboxylated MGP decreasing vascular calcification. Multiple studies have demonstrated that vitamin D and K have a synergistic effect, and the diagram above outlines how they can be used in combination to prevent fractures in both healthy and osteoporotic subjects, as well as reducing vascular calcification. [47].

Currently, Yi-Chou Hou et al.’s review coincides with the study carried out by Jiang et al., as vitamin K_2_ has been proven to prevent vascular calcification. Furthermore, the review expands on vitamin D’s ability to stimulate osteocalcin synthesis as well as the binding of VKDPs to osteocalcin. This promotes osteogenesis through the transformation of osteoblasts to osteocytes. This study evaluated the benefits of utilising vitamin D and K_2_ concordantly to improve insulin sensitivity and vascular thickness in diabetic subjects. This review further reinforced that vitamin K_2_ may be used in isolation for treatment for many pathologies, and when used alongside vitamin D, it can reverse calcification [47]. This process is illustrated in Figure 5. 

Yi-Chou Hou et al.’s review thoroughly investigated the relation between vitamin D and vitamin K_2_. Compared to the extensive research into the effects of vitamin K_2_ on bone and vascular calcification, the use for the treatment for pathologies of the parathyroid is somewhat unknown. In 2003, Nakashima et al. acknowledged that patients receiving haemodialysis had significantly reduced serum level of vitamin K, which led to an increased incidence of fractures. This article studied how vitamin K_2_ can be used as treatment for haemodialysis patients with a low PTH level through analysing bone biomarkers. After 12 months, there was no significant increase in the serum levels of MK-4, but the concentration of carboxylated OC levels had increased. There was also an evident increase in the concentration of ALP 3 months after the start of the study, coinciding with Jian et al.’s findings [48]. While vitamin K_2_ has demonstrated the ability to change the concentration of these bone biomarkers, the study revealed that it has no impact on the levels of PTH, calcium or serum phosphate, and therefore, it cannot be used in isolation for parathyroid conditions. Due to its ability to enhance the carboxylation of VKDPs such as MGP in blood vessels, vitamin K_2_ has the potential for use in cases of hyperparathyroidism, and may be able to prevent the associated hypercalcaemia. In theory, an increase in dosage could result in more osteoclastic apoptosis as well as preventing the calcification of vessels. 

In 2018, the effects of vitamin K_2_ on the synthesis of bile and homeostasis of glucose was investigated by Sultana et al. The pregnane X receptor (PXR), sometimes referred to as SXR, is a subtype of nuclear receptors, which, when activated by ligands, leads to the transcription of proteins. Earlier reports suggested that MK-4 directly binds to the PXR, helping to promote related compounds to act similarly. This was further investigated in this study, through using two types of rats: one group of wild-type rats, and another with human genetic coding sequence for the PXR encoded into its DNA. Analysing the results, there was no significant difference between MK-4 content in the livers of both groups of rats. In contrast to the MK-4 having no effect on the wild-type rats, the humanised mice showed a significant increase in the mRNA levels of numerous proteins such as ATP and cytochrome P450. At low MK-4 doses, there was a reduction in the mRNA levels of the genes Cyp7a1 and Cyp8b1. The glucose homeostasis gene Slc2a5 concurrently showed a marked decrease in mRNA level, this could indicate that MK-4 can affect the metabolism of administered drugs in patients being treated with the vitamin. This study also questioned whether, due to its effects in reducing bile synthesis, MK-4 could be used alongside treatment for cholestasis [49].

He Ma et al. analysed the effect of varying dosages of vitamin K_2_ on the action of the VKDPs, GGCX and MGP in regulating sperm maturation. Prior to this study, it was known that calcium plays a crucial role in enabling the maturation of sperm both in the testes and post ejaculation in the seminal fluid. Using PCR techniques, the researchers assessed the levels of mRNAs of these VKDPs in wild rats. The mRNA levels of MGP were 10-fold higher in the epididymis than in the testes. Using warfarin to inhibit the enzymes resulted in a reduced sperm count and reduction in percentage of total and motile spermatozoa. As previously stated, warfarin inhibits the action of vitamin K_2_ to act as a co-factor in the γ-carboxylation of proteins. Therefore, this study exemplified the risk of an inadequate level of vitamin K on sperm maturation [32]. 

There is still a wide range of unexplored benefits of vitamin K_2_ that could be investigated, such as reducing the incidence of fractures in patients with cerebral palsy. An evidence-based review carried out by Cohen et al. assessed literature which investigated the bone strength of subjects aged 3 to 21 years old who were diagnosed with cerebral palsy. The study concluded that there was a significant decrease in the BMD of these patients, putting them at higher risk of fracturing their bones [50]. Furthermore, this review acknowledges the benefits of vitamin K_2_, as well as looking at the effects of vitamin D [50]. This furthers the results of Miyake et al., revealing the synergistic effects of these two vitamins on the levels of bone biomarkers, which gives the possibility of their use in management of cerebral palsy [43]. 

## 5. Conclusions

Vitamin K_2_ has now established a compelling platform within the scientific community as a compound that expresses beneficial properties that can be utilised in the medical field. Currently, the mainstay of research has been focused on its effects on bone metabolism as this was initially where the Japanese research began. There have been many studies which have concluded a significant increase in the serum levels of OC in osteoporotic subjects. When healthy subjects were given the same dosage of vitamin K_2_, there was still a significant rise in the OC serum level; however, this did not in turn cause a significant reduction in the incidence of fracture as found in studies on osteoporotic patients. 

Specifically reviewing the effects of vitamin K_2_ on BMD, the overall consensus was that there is an initial increase in BMD, whether it be in the hip or the femur; however, after long term daily administration, this returned to baseline, as shown in the study by Shiomi et al. [37]. It is possible that vitamin K_2_ can only benefit the BMD of those with pathological bone conditions, including osteoporosis, as suggested by the results of the study by Kobayashi et al. [39]. This showed that there in fact is a reduction in the incidence of fractures, but only at mild levels of osteoporosis. 

Vitamin D has displayed a clear role in both stimulating the production of VKDPs and aiding vitamin K_2_ to carboxylate said proteins. Studies have shown that when given in combination, they exhibit a synergistic effect on bone metabolism, sperm maturation, bile synthesis, and vascular calcification. However, the studies currently available are sparce; therefore, further investigation into these areas is required. The presence of high levels of mRNA for GGCX and MGP in the testes indicates that vitamin K_2_ could have unknown effects on the body; this ought to be investigated further to confirm the conclusions of studies such as that by Miyake et al., who showed increases in carboxylated OC in their subjects [43]. 

Vitamin K_2_ possesses the ability to prevent and reverse vascular calcification, as demonstrated by Jiang et al. [12]. If these trials were reproduced on a human sample, it could allow for radical changes in the current treatment of cardiovascular disease. In conjunction with other anti-platelets and blood thinning medication, the use of vitamin K_2_ to prevent vascular calcification needs to be further investigated to confirm whether it is beneficial and what dosages are required to produce these effects.

In comparison to the quantities of research on the effect of vitamin K_2_ on bone metabolism and vascular calcification, to name a couple, there has been little research on its ability to be used for other diseases, such as hyperparathyroidism and cerebral palsy. Nakashima et al. showed that serum carboxylated OC concentrations had increased after 12 months of treatment of MK-4 [48]. Further research carried out using other vitamers, such as MK-7, would permit a comparison to existing results and a definitive conclusion on its use to treat hypoparathyroidism could be made. Due to its properties in reducing vascular calcification, there is potential for hyperparathyroidism to also be treated with vitamin K_2_ due to the prevention of calcification. Additionally, Cohen et al. identified low BMD in cerebral palsy subjects, concluding that due to the properties exhibited by vitamin K_2_, future research could confirm its use in treatment [50].

To date, the dosage of vitamin K_2_ varies across different countries, with Japan recommending 150 micrograms to its population [45]. Many studies showed that the effects of vitamin K_2_ increased in a dose-dependent manner; thus, the question remains which dosage will correlate to the maximal effect whilst avoiding over-compensation. MK-7 is known to be of greater benefit than MK-4 due to its longer half-life; therefore, finding the optimal dosage of MK-7 to induce beneficial effects in the body would prove insightful. 

This report has briefly discussed the biochemistry of vitamin K_2_ and the metabolism of calcium before going onto to explore the current literature available that combines the two topics together. In doing this, it has been established that in the past two decades, the research on the topic has developed remarkably. Vitamin K_2_ has proven to directly benefit osteoporotic patients, primarily through an increase in the γ-carboxylation of VKDPs, such as MGP and GGCX, and enzymes such as ALP, which are associated with bone mineralisation, sperm maturation, vascular calcification and bile acid synthesis, among other processes. However, more research into this topic is required to establish the direct effect of vitamin K_2_ in healthy individuals to ascertain whether it can be used as prophylaxis or treatment in patients exhibiting pathologies such as diabetes, osteoporosis, cerebral palsy and parathyroid disorders. 

## Data Availability

No new data were created or analysed in this study. Data sharing is not applicable to this article.

## Abbreviations

7-dehydrocholesterol	7-DHC
Menaquinone-n	MK-n
Triglyceride-rich lipoproteins	TRLs
High-density lipoproteins	HDLs
Low-density lipoproteins	LDLs
Uncarboxylated osteocalcin	ucOC
Heparan sulphate proteoglycan	HSPG
Vitamin K-dependent proteins	VKDP
Matrix Gla protein	MGA
Growth arrest-specific protein 6	Gas6
Osteocalcin	OC
Osteopontin	OP
Gla-rich protein	GRP
Periostin-like factor	PLF
Proline-rich Gla protein	PRGP
Transmembrane Gla protein	TMG
γ-glutamyl carboxylase	GGCX
vitamin K-hydroquinone	KH_2_
vitamin K-epoxide	KO
vitamin K-oxidoreductase	VKOR
Parathyroid hormone	PTH
Primary hyperparathyroidism	PHP
Gastrointestinal tract	GIT
Bone mineral density	BMD
Smooth muscle cells	SMC
Dephosphyryated-uncarboxylated MGP	dp-ucMGP
Undercarboxylated MGP	ucMGP
Chronic kidney disease	CKD
Cationic sperm	CatSper
United Kingdom	UK
International Osteoporosis Foundation	IOF
Dual energy X-ray absorptiometry	DXA
Bone mineral content	BMC
Alkaline phosphatase	ALP
Pregane X receptor	PXR
Vascular smooth muscle cell	VSMC
